# Inhibition of insulin-degrading enzyme in human neurons promotes amyloid-β deposition

**DOI:** 10.1042/NS20230016

**Published:** 2023-10-03

**Authors:** Helen A. Rowland, Samuel R. Moxon, Nicola J. Corbett, Kelsey Hanson, Kate Fisher, Katherine A.B. Kellett, Nigel M. Hooper

**Affiliations:** 1 Division of Neuroscience, School of Biological Sciences, Faculty of Biology, Medicine and Health, University of Manchester, M13 9PT, Manchester, U.K.; 2 Geoffrey Jefferson Brain Research Centre, Manchester Academic Health Science Centre, Northern Care Alliance and University of Manchester, Manchester, U.K.

**Keywords:** Alzheimers disease, amyloid beta, insulin-degrading enzyme, metalloproteases, neprilysin, neurons

## Abstract

Alzheimer’s disease (AD) is characterised by the aggregation and deposition of amyloid-β (Aβ) peptides in the human brain. In age-related late-onset AD, deficient degradation and clearance, rather than enhanced production, of Aβ contributes to disease pathology. In the present study, we assessed the contribution of the two key Aβ-degrading zinc metalloproteases, insulin-degrading enzyme (IDE) and neprilysin (NEP), to Aβ degradation in human induced pluripotent stem cell (iPSC)-derived cortical neurons. Using an Aβ fluorescence polarisation assay, inhibition of IDE but not of NEP, blocked the degradation of Aβ by human neurons. When the neurons were grown in a 3D extracellular matrix to visualise Aβ deposition, inhibition of IDE but not NEP, increased the number of Aβ deposits. The resulting Aβ deposits were stained with the conformation-dependent, anti-amyloid antibodies A11 and OC that recognise Aβ aggregates in the human AD brain. Inhibition of the Aβ-forming β-secretase prevented the formation of the IDE-inhibited Aβ deposits. These data indicate that inhibition of IDE in live human neurons grown in a 3D matrix increased the deposition of Aβ derived from the proteolytic cleavage of the amyloid precursor protein. This work has implications for strategies aimed at enhancing IDE activity to promote Aβ degradation in AD.

## Introduction

The pathogenesis of Alzheimer’s disease (AD) is characterised by the aggregation and deposition of amyloid-β (Aβ) peptides in the brain. Aβ is proteolytically cleaved from the larger amyloid precursor protein (APP) through the sequential action of the β-secretase, β-site APP cleaving enzyme-1 (BACE1), and the presenilin-containing γ-secretase complex [1]. In familial forms of AD, mutations in APP, presenilin 1 or presenilin 2 give rise to increased production of Aβ and/or an increase in the ratio of Aβ1-42:Aβ1-40 [2–4]. However, in age-related late-onset sporadic AD, which accounts for >95% of all AD cases, deficient clearance of Aβ contributes to disease pathology [5–7].

Aβ clearance is brought about through extracellular and intracellular proteolytic degradation and efflux of extracellular Aβ into the blood via the blood–brain barrier [8,9]. Several proteases have been implicated in the proteolytic degradation of Aβ, including angiotensin-converting enzyme, endothelin-converting enzymes (ECE1 and ECE2), insulin-degrading enzyme (IDE), matrix metalloproteases and neprilysin (NEP) (reviewed in [8,10–12]). Various studies have shown that IDE and NEP are the major Aβ-degrading proteases in rodents. Mice with inactivation of the genes encoding IDE [13,14] and NEP [15] had a moderate (1.5- to 2-fold) increase in endogenous Aβ. Inhibition of NEP significantly reduced the degradation of Aβ in the rat hippocampus [16] and up-regulation of NEP by somatostatin reduced brain Aβ levels [17]. In primary rat cortical neurons extracellular levels of Aβ were regulated via proteolysis by IDE [18] and a natural partial loss-of-function mutation in IDE led to impaired Aβ degradation in a rodent model [19]. However, no study has yet investigated the contribution of IDE and NEP to Aβ degradation in human neurons.

In AD, Aβ is primarily deposited outside neurons in the extracellular matrix (ECM). In the vast majority of cell-based studies on Aβ metabolism, the cells are grown in 2D on the surface of plastic dishes in the absence of an ECM. The lack of an ECM, together with frequent media changes, prevents the deposition of Aβ. However, embedding cells within a 3D ECM allows the visualisation of Aβ deposits as diffusion of the peptide is limited [20–22].

Here, we investigated the contribution of IDE and NEP to Aβ degradation by human induced pluripotent stem cell (iPSC)-derived cortical neurons using an Aβ fluorescence polarisation assay [23]. In addition, we exploited the observation that Aβ deposition can be visualised when neurons are grown in a 3D ECM, to determine the impact of inhibition of IDE and NEP on Aβ metabolism in live human neurons.

## Experimental

### Culture and differentiation of iPSC-derived cortical human neurons

The iPSC lines OX1-19 (obtained from S. Cowley, University of Oxford) [24] and SBAD03-05 (obtained from StemBANCC) [25] were maintained on Matrigel (Corning #354277) in mTeSR1 medium (StemCell Technologies #85850) at 37°C with 5% CO_2_. iPSCs were differentiated to cortical neurons via dual SMAD inhibition as previously described [26]. At day 0 confluent iPSCs were treated with 1 μM dorsomorphin (Tocris #30-931-0) and 10 μM SB431452 (Tocris #1614) in neural maintenance media (NMM) (500 ml DMEM/F-12 Glutamax (Life Technologies #10565018), 500 ml Neurobasal (Life Technologies #12348017), 5 ml N-2 supplement (Life Technologies #17502048), 10 ml B-27 supplement (Life Technologies #17504044), 2.5 μg/ml insulin (Sigma #I9278), 1 mM L-glutamine (Life Technologies #25030081), 0.5X non-essential amino acids (Life Technologies #11140050), 50 μM 2-mercaptoethanol (Life Technologies #125470010), 50 U/ml penicillin/streptomycin (Life Technologies #15140122), 0.5 mM sodium pyruvate (Sigma #S8636)) for 10–11 days before disassociation with dispase (Stemcell Technologies #07923) and replated on laminin (Sigma #L2020). From this point forward, cells were incubated in NMM with media changes every 2–3 days. Between days 12 and 17 of neuronal induction, neural rosettes formed. FGF2 (20 ng/ml) (Peprotech #100-18B) was added for 2–4 days to help promote a neural fate. Cells were expanded and passaged a further 2–3 times using dispase and STEMdiff Rosette Selection Reagent (Stemcell Technologies #05832) to expand neuronal cells and remove unwanted cell types. Between days 27 and 31 after neuronal induction, neural progenitor cells (NPCs) were passaged using Accutase (Stemcell Technologies #07920). For routine 2D cell culture, NPCs were plated at approximately 26,000 cells per cm^2^ on plates coated with both poly-L-ornithine (Sigma #P4957) and laminin and matured to neurons. Experiments were performed when neurons reached day 60–80. All images shown are from the OX1-19 cell line, unless otherwise indicated.

### Immunofluorescence microscopy

OX1-19 iPSC-derived neurons were cultured to day 80 on coverslips and fixed in 4% paraformaldehyde (PFA). Coverslips were blocked in 10% donkey serum (Sigma #D9663). Antibodies against MAP2 (Abcam #ab92434) and βIII-Tubulin (Abcam #ab18207) were used. Coverslips were then incubated in Alexa-fluor fluorescent secondary antibodies (ThermoFisher), mounted on slides using prolong gold containing 4′,6′-diamidino-2-phenylindole (DAPI) (358nm absorbance) (Cell Signalling Technology #8961) and imaged on an EVOS® FL Cell Imaging System (ThermoFisher).

### Membrane potential assay

Membrane potential was measured using the FLIPR® assay according to the manufacturer’s protocol (Molecular Devices #R8128). iPSC-derived neurons were differentiated in a black clear-bottom 96-well plate until day 60 or day 80 then incubated in the RED indicator dye for 30 min in 5% CO_2_ at 37°C as per the manufacturer’s protocol. Plates were then placed into the FlexStation™ instrument (Molecular devices) to measure fluorescence (excitation: 530 nm, emission: 565 nm), with 60 mM KCl added 19 s into the recording and fluorescence measured for a total of 90 s.

### Aβ degradation assay

Assessment of Aβ degradation in cell lysates was based on a previously published fluorescence polarisation assay [23]. Day 80 iPSC-derived neurons were washed once with phosphate-buffered saline (PBS) (Sigma #D8662) before lysis with 150 mM sodium chloride, 1% (v/v) Nonidet-P40, 50 mM Tris/HCl, pH 8.0) for 25 min on ice. The solution was then centrifuged at 14000 × ***g*** for 10 min at 4°C and the clarified lysate recovered by taking 85% of the supernatant. Lysates containing 20 µg of protein were loaded into black clear-bottom 96-well plates. Samples were kept on ice and contained no protease or phosphatase inhibitors. Aβ1-40-Lys(LC-biotin)-NH_2_, FAM-labelled (Anaspec #AS-61962-01) was added to give a final concentration of 500 nM per well. DMEM was used to make a final volume of 100 µl. Starting fluorescence was measured (excitation: 485 nm, emission: 508 nm) on a Synergy HT plate reader (Biotek). The plate was then incubated for 4 h at 37°C and the fluorescence measured again for confirmation of baseline. Magnetic Dynabeads (2µl MyOne Streptavidin T1, ThermoFisher #65601) were added to each well and the plate placed on a shaker for 30 min. The plate was then transferred to a magnetic platform (ThermoFisher) and the magnetic beads pulled to one side for 5 min. The supernatant was transferred to new wells and the final fluorescence value measured. Degradation was calculated by taking the final fluorescence value over the starting fluorescence value. For experiments with recombinant IDE and NEP the following amounts were used: 25 ng NEP (BioTechne #1182-ZNC-010 (lot:RXD0217041)) or 25 ng IDE (Bio-Techne #2496-ZN-010 (lot:NSA1016031)). To inhibit proteolytic activity the following inhibitors were added at the indicated final concentration: 1 mM 1,10-phenanthroline (Sigma #131377), 100 µM phosphoramidon (Tocris Bioscience #6333), 10 µM 6bK (Tocris Biosciences #5402), 10 µM ML345 (Sigma #SML1117), 100 µM insulin. Inhibitors and proteases were preincubated for 30 min at 37°C prior to the addition of FAβB.

### IDE and NEP enzymatic assays

IDE activity was measured in iPSC-derived neuron lysates using the SensoLyte 520 IDE Activity Fluorometric Assay Kit (Anaspec #AS-72231) in the absence or presence of 10 μM ML345 as per the manufacturer’s instructions. IDE specific activity was taken as the activity inhibited by ML345. NEP activity was measured in iPSC-derived neuron lysates using the SensoLyte 520 NEP Activity Fluorometric Assay Kit (Anaspec #AS-72223) in the absence or presence of 100 μM phosphoramidon as per the manufacturer’s instructions. NEP specific activity was taken as the activity inhibited by phosphoramidon.

### Aβ deposition assay using 3D Matrigel cultures

For Aβ deposition experiments, day 40 NPCs from either OX1-19 or SBAD03-05 lines were suspended at approximately 500,000 cells per ml in 5% Matrigel diluted with NMM. 3D Matrigel cultures were then plated at 500 μl per well into 12-well plates and incubated at 37°C for 2 h to embed the neurons. A further 1 ml of media was then added on top of the 3D Matrigel culture and the neurons grown with media changes every 2–3 days. iPSC-derived neuron cultures were maintained for 21 days before analysis. For inhibition experiments, neurons were incubated with either phosphoramidon (10 μM), ML345 (10 μM) or βIV (10 μM) (Merck #565788) supplemented into the media for the duration of the experiment. To evaluate Aβ deposition embedded neurons were fixed in 4% PFA for 20 min, washed with PBS containing 50 mM NH_4_Cl and then permeabilised in 0.2% Triton X-100 for 15 min. Neurons were then blocked for 2 h in 10% donkey serum before being incubated overnight at 4°C with primary antibodies against βIII-Tubulin and Aβ (4G8) (Biolegend #SIG-39220) or with A11 (Invitrogen #AHB0052) or OC (Millipore #AB2286) conformational antibodies. All primary and secondary antibodies were used at a working concentration of 1:500. Neurons were washed with PBS before incubation with Alexafluor fluorescent secondary antibodies (ThermoFisher #A-21202, ThermoFisher #A-21207). Neurons were imaged on an EVOS® FL Cell Imaging System with six randomised images taken from each well. The Aβ deposits were analysed using ImageJ (National Institutes of Health) and the total amount of 4G8 positive Aβ deposits present in each image quantified. To prevent unconscious bias during the imaging process, the fluorescent channel for βIII tubulin staining was used to locate neuronal populations. The channels corresponding to Aβ staining were not observed until after an area was selected for imaging and the location of the image was not changed once identified.

### Statistical analysis

Statistical tests were performed using SPSS (IBM, v23) and GraphPad Prism (GraphPad, v8) and statistical significance was set at *P*<0.05. For comparisons of data with multiple groups a Brown–Forsythe test was used to determine whether the standard deviations (SDs) of group data were significantly different. As SDs were not significantly different a two-tailed ordinary one-way ANOVA test was used with a Dunnett’s post-hoc analysis to compare experimental groups to control. For comparison of data with only two groups a Welch’s *t*-test was used to account for unequal SDs between groups. Information for each analysis and *n* numbers for each experiment are given in the relevant figure legend.

## Results

**Figure 1:**
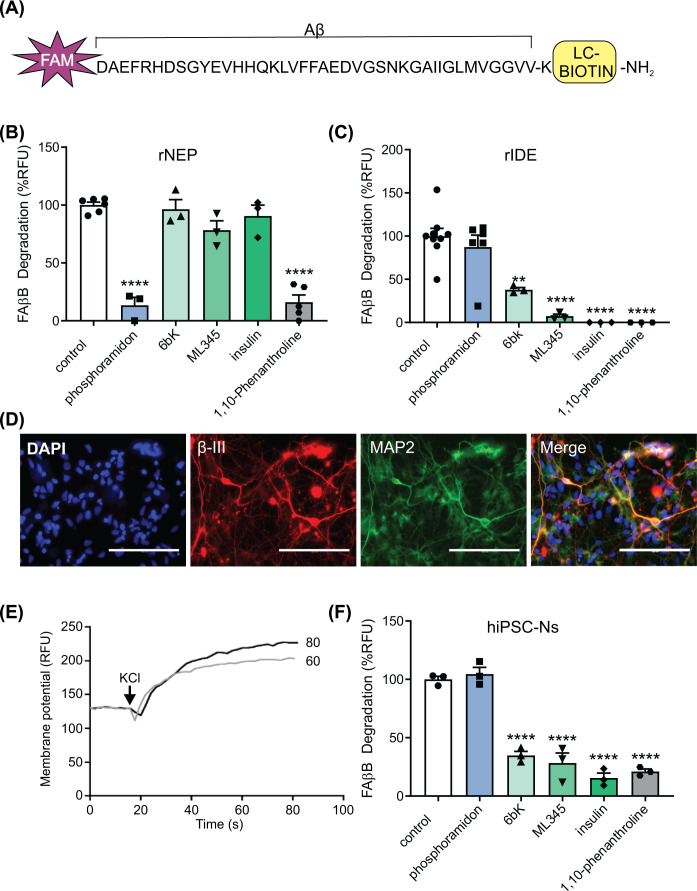
IDE degrades Aβ in iPSC-derived neurons (**A**) Schematic diagram of the FAM-Aβ-biotin substrate used in the fluorescence polarisation assay. Incubation of the Aβ substrate (500 nM) with (**B**) 25 ng recombinant NEP (rNEP) and (**C**) 25 ng recombinant IDE (rIDE) in the absence or presence of the general metalloprotease inhibitor 1,10-phenanthroline (1 mM), the NEP inhibitor phosphoramidon (100 μM) or the IDE inhibitors 6bK (10 μM), ML345 (10 μM) and insulin (100 μM). NEP and IDE were pre-incubated with the inhibitors for 30 min at 37°C before addition of the FAM-Aβ-biotin substrate and a further incubation for 4 h at 37°C. The cleaved substrate was separated and the fluorescence measured as described in the Experimental section. Data shown as mean ± SEM, *n* = 3 experimental repeats. (**D**) OX1-19 iPSCs were differentiated to cortical neurons and neuronal identity was confirmed at day 80 by identification of neuronal markers using immunofluorescence microscopy. Representative images demonstrate staining for the neuronal markers MAP2 and β-III tubulin (β-III). Nuclei were stained with DAPI, scale bar represents 200 µm. (**E**) Membrane potential was measured in neurons using the FLIPR® Membrane Potential Assay Kit. Representative traces shown for neurons at day 60 and day 80 of differentiation. Experiments were repeated in three independent cell preparations. (**F**) Incubation of the Aβ substrate with human OX1-19 iPSC-derived neurons (hiPSC-Ns) in the absence or presence of the indicated inhibitors. *n* = 3 neuronal inductions (three technical repeats were performed per neuronal induction). **P<0.005, ****P<0.0001 using one-way ANOVA with Dunnett’s multiple comparisons test to compared with control.

A method to measure Aβ degradation was established based on a previously described fluorescence polarization assay that uses Aβ1-40 tagged at its N-terminus with carboxyfluorescein (FAM) and at its C-terminus with biotin [23] (Figure 1A). Proteases in the experimental sample cleave the tagged Aβ. Streptavidin-coated dynabeads are then added which bind to the biotin tag and can pull down uncleaved peptide or C-terminal fragments. Cleaved N-terminal fragments of Aβ remain in the supernatant and their fluorescence can be measured due to the FAM label at the N-terminus. Addition of the FAM and biotin had no observable impact on the metabolism of the Aβ by NEP and IDE and the modified peptide had a similar inhibition profile as wild-type Aβ in biological samples [23]. Initially, the assay was validated using recombinant forms of IDE and NEP. Both recombinant NEP (Figure 1B) and recombinant IDE (Figure 1C) degraded the Aβ. To investigate subsequently which of the two proteases was contributing to Aβ degradation in neurons, selective inhibitors were profiled. The degradation of Aβ by recombinant NEP was inhibited by 1,10-phenanthroline (a general metalloprotease inhibitor) and by phosphoramidon which inhibits selectively NEP [27] but not by the IDE selective inhibitors 6bk and ML345 [28,29] (Figure 1B). The degradation of Aβ by recombinant IDE was also inhibited by 1,10-phenanthroline and by 6bK and ML345, but not by phosphoramidon (Figure 1C). Insulin was used as a competitive inhibitor of IDE as the protease has greater affinity for insulin compared with Aβ (*K*_m_ 85 nM and 25 μM, respectively) [30]. Insulin also inhibited the degradation of Aβ by IDE (Figure 1C).

The selective protease inhibitors were then used to determine the contribution of NEP and IDE to Aβ degradation in iPSC-derived human cortical neurons. The OX1-19 iPSC line used in the present study was derived from an individual without AD and does not have mutations in APP or the presenilins. The OX1-19 iPSCs were differentiated into neurons and matured for 60–80 days following an established protocol [26]. At day 80, iPSC-derived neurons were positive for the neuronal markers MAP2 and βIII-tubulin (Figure 1D). At both day 60 and day 80, the neurons depolarized in response to KCl (Figure 1E), demonstrating that functionally active cortical neurons had been obtained. In neuronal lysates both IDE and NEP activities were detected using selective fluorescent peptide substrates in the absence and presence of ML345 or phosphoramidon, respectively (see Experimental section for details). From the assays with these selective fluorescent substrates, IDE specific activity was determined as 4.41 ± 0.50 nM 5-FAM/min/mg protein, and NEP specific activity as 0.63 ± 0.26 nM 5-FAM/min/mg protein. Aβ was degraded by the neuron lysates and this degradation was inhibited by all three of the IDE inhibitors (6bk, ML345 and insulin), as well as by 1,10-phenanthroline, but not by the NEP inhibitor phosphoramidon (Figure 1F). These data indicate that although both IDE and NEP activities are present in the iPSC-derived neurons, only inhibition of IDE reduces Aβ degradation.

**Figure 2:**
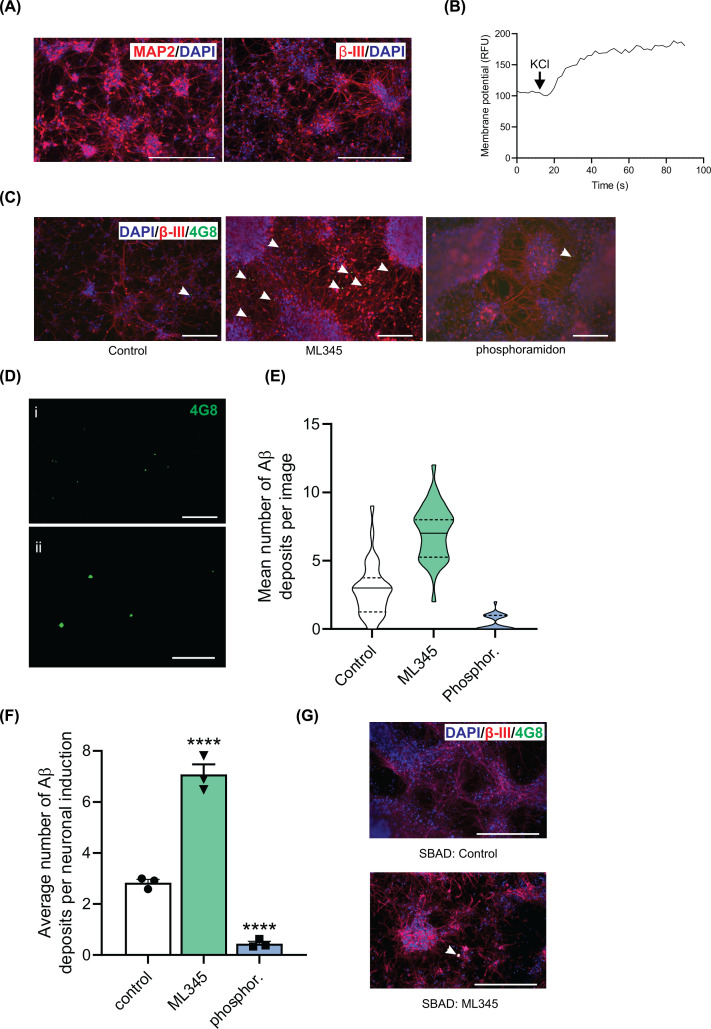
Inhibition of IDE in human neurons increases Aβ deposition in a 3D matrix (**A**) OX1-19 iPSC-derived neurons seeded into 3D matrigel cultures were imaged for neuronal markers MAP2 and β-III tubulin, scale bar = 200 μm. (**B**) Neuronal membrane potential was measured in neurons using the FLIPR® Membrane Potential Assay Kit. Representative trace of membrane potential from neurons in 3D matrigel cultures. Day 60 OX1-19 neurons were cultured in a 3D Matrigel matrix for 21 days in the absence or presence of the IDE inhibitor, ML345 (10 μM) or the NEP inhibitor, phosphoramidon (10 μM). (**C**) Representative images of OX1-19 neurons following treatment without or with ML345 or phosphoramidon using immunofluorescence microscopy to determine the number of Aβ deposits; composite images of βIII-tubulin and Aβ are shown and Aβ staining (with antibody 4G8) is highlighted by the arrowheads, scale bar = 200 µm. (**D**) Representative images of OX1-19 neurons following ML345 treatment showing only the Aβ deposits stained with antibody 4G8 with (i) being the same field of view as shown in (**C**), scale bar = 200 µm and (ii) a larger magnification image from a separate field of view, scale bar = 100 µm. (**E**) The number of Aβ deposits in each image was quantified and the spread of data from images for each condition are shown by the violin plot. Data are shown with median (solid line) and quartiles (dashed line) for 36 data points for control and ML345 and 25 data points for phosphoramidon. (**F**) The number of Aβ deposits was then averaged for each neuronal induction for each condition and statistical analysis performed. Data shown as mean ± SEM, *n* = 3 neuronal inductions (three technical repeats were performed per neuronal induction). ****P<0.0001 using an ordinary one-way ANOVA test with Dunnett’s multiple comparisons test to compared with control. (**G**) Representative images taken for the analysis of Aβ deposition in day 60 SBAD neurons cultured in a 3D Matrigel matrix for 21 days in the absence or presence of the IDE inhibitor, ML345 (10 μM) using immunofluorescence microscopy, scale bar = 200 µm.

To investigate the contribution of IDE and NEP to Aβ degradation in live cells, iPSC-derived neurons were encapsulated in Matrigel to enable the formation and detection of Aβ deposits [20]. The encapsulated neurons expressed the neuronal markers MAP2 and βIII-tubulin and depolarized in response to KCl (Figure 2A,B) similarly to the neurons in 2D (Figure 1D,E), indicating that encapsulation in Matrigel does not adversely affect neuronal differentiation and function. The encapsulated neurons were then incubated with either the IDE inhibitor ML345 or the NEP inhibitor phosphoramidon for 21 days and Aβ deposits detected with the anti-Aβ antibody 4G8. Although some Aβ deposits were visible in the neurons encapsulated in Matrigel, the number of deposits was significantly increased in the presence of the IDE inhibitor ML345 (Figure 2C–F). In contrast, no such increase in the number of Aβ deposits occurred in the presence of the NEP inhibitor phosphoramidon (Figure 2C,E,F). Instead, phosphoramidon treatment led to a decrease in the number of Aβ deposits. In the absence of inhibitors, the Aβ deposits ranged in size from 1 to 550 μm^2^ with the majority (84%) of aggregates in the range 1–150 μm^2^ (mean deposit size 78 μm^2^, median deposit size 50 μm^2^). Inhibition of IDE also increased Aβ deposition in neurons derived from a second, independent iPSC line, SBAD03-05 [25,31] (Figure 2G).

**Figure 3:**
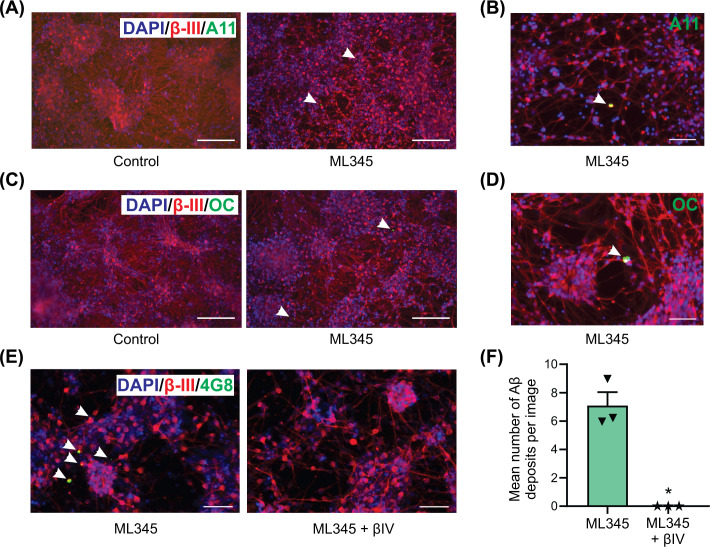
Aβ deposits are composed of oligomeric species and their formation is blocked by a β-secretase inhibitor Day 60 OX1-19 neurons were cultured in a 3D Matrigel matrix for 21 days in the absence or presence of the IDE inhibitor, ML345. Representative images taken for the analysis of Aβ species by immunofluorescence microscopy with conformational antibodies to detect (**A**) pre-fibrillar Aβ oligomers with the A11 antibody, with a higher magnification image shown in (**B,C**) fibrillary Aβ oligomers with the OC antibody, with a higher magnification image shown in (**D**). (**E**) Representative images of day 60 OX1-19 neurons cultured in a 3D Matrigel matrix for 21 days in the presence of the IDE inhibitor, ML345, with or without the BACE1 inhibitor βIV. (**F**) Quantification of Aβ deposits in OX1-19 neurons following treatment with either ML345 only or with ML345 and βIV. Scale bar in A and C = 200 µm, scale bar in B, D and E = 100 µm. Data shown as mean ± SEM, *n* = 3 independent cell preparations from the same neuronal differentiation *P<0.05 using a Welch’s *t*-test.

To characterise the IDE-inhibited Aβ deposits, we utilised the conformation-dependent, anti-amyloid antibodies A11 and OC [32,33]. The Aβ deposits in the neurons were stained with both antibodies indicating the presence of both pre-fibrillar (A11 positive) and fibrillar (OC positive) aggregated species (Figure 3A–D). Blocking Aβ formation with the BACE1 inhibitor βIV prevented the formation of the Aβ deposits even in the presence of the IDE inhibitor ML345 (Figure 3E,F), confirming that these are deposits of Aβ derived from proteolytic cleavage of APP.

## Discussion

In late-onset sporadic AD, deficits in the degradation and clearance of Aβ, rather than enhanced production, underlie the increase in Aβ [5–7]. The Aβ peptides aggregate, forming a range of oligomeric species that further aggregate into protofibrils and fibrils, and are deposited in the brain in the form of amyloid plaques [34]. Here, we show that inhibition of the Aβ-degrading enzyme IDE increases Aβ deposition when iPSC-derived cortical neurons are encapsulated in a 3D ECM, with the deposited Aβ having immunological characteristics of Aβ plaques found in post-mortem human brain tissue.

IDE is a zinc-dependent protease located in the cytosol, peroxisomes, mitochondria and at the cell surface (reviewed in [35]). Although IDE lacks a canonical signal peptide sequence, it is secreted from cells and is present in the extracellular medium [36]. Our study does not allow us to determine whether IDE is acting on the Aβ in the extracellular milieu or following internalisation of the peptide. The IDE inhibitors 6bk, ML345 and insulin inhibit IDE through different mechanisms: 6bK is a synthetic macrocycle that engages a binding pocket away from the catalytic site and is remarkably selective for IDE over a number of other zinc metalloproteases including NEP [28], the benzoisothiazolidone ML345 targets a specific reactive cysteine residue in IDE that is not present in NEP and the majority of other zinc metalloproteases [29], and insulin, being a substrate for IDE, acts as a competitive inhibitor at high concentrations [30]. As these three compounds inhibit IDE through separate mechanisms this provides confirmation that the Aβ degrading activity observed in the iPSC-derived neurons is due to IDE. The possibility of these inhibitors having off-target effects cannot be ruled out, and future studies using genetic knockdown approaches could be used to confirm the results using the selective protease inhibitors. However, this major role for IDE in Aβ degradation in human neurons is consistent with previous reports showing that IDE is the major protease responsible for Aβ degradation in human hippocampal lysates and cerebrospinal fluid [37,38].

The selectivity of 6bk and ML345 for IDE over the structurally and mechanistically distinct NEP [28,29], together with the lack of effect of the NEP inhibitor phosphoramidon to increase the number of Aβ deposits, indicates that NEP does not play a significant role in Aβ degradation in human iPSC-derived cortical neurons. Interestingly, phosphoramidon actually decreased the number of Aβ deposits in the neuron culture. IDE is activated by a number of biologically active peptides, including bradykinin, β-endorphin and the dynorphins, and of particular relevance here is the observation that dynorphin B-9 activated IDE and increased Aβ hydrolysis by 2.5-fold [39]. This modulatory effect is due to an allosteric mechanism between the subunits in the IDE dimer; binding of the biologically active peptide to the active site of one IDE subunit induces an enhancement of IDE proteolytic activity towards Aβ by the other subunit [40]. NEP cleaves numerous biologically active peptides including bradykinin and the dynorphins [41]. Thus, a potential explanation for the observation that phosphoramidon reduces the number of Aβ deposits in the neurons is that inhibition of NEP increases the levels of bradykinin, dynorphins or other peptides that allosterically activate IDE resulting in increased hydrolysis of Aβ and reduced deposition of intact peptide. This phenomenon may be exacerbated when the neurons are grown in a 3D matrix in which the small biologically active peptides act locally within the ECM rather than being dispersed into the cell media when the neurons are grown in 2D. ECE1 and ECE2 have also been implicated in the degradation of Aβ [42], but as these two proteases are also inhibited by phosphoramidon [43], it is unlikely that either of these proteases have a significant role in Aβ degradation in the human iPSC-derived cortical neurons. Another metalloprotease that can cleave Aβ *in vitro* is angiotensin-converting enzyme 1 [44], although it appears to have a limited role in Aβ metabolism *in vivo* [45]. The possibility of these or other proteases contributing to Aβ metabolism in the iPSC-derived neurons cannot be completely ruled out, although as the IDE inhibitors reduced Aβ degradation by >65%, IDE is clearly the predominant Aβ-degrading protease in this system.

Using fluorogenic substrates and selective inhibitors, both IDE and NEP activities were detected in the iPSC-derived cortical neurons. There was a higher specific activity for IDE compared to NEP, although as different substrates had to be used for the two proteases, a direct comparison of activities is difficult. According to the Human Protein Atlas (www.proteinatlas.org), IDE mRNA levels are higher in the hippocampus (3.4 nTPM (highest normalized expression)) and cerebral cortex (4.2 nTPM), than NEP mRNA levels (0.1 nTPM and1.9 nTPM, respectively). Whether these differences in transcript levels equate to similar differences in protein levels remains to be determined, but the potentially higher IDE than NEP activity in human iPSC-derived neurons would be consistent with the respective brain mRNA levels.

The Aβ deposits observed here when neurons were grown in 3D ECM are similar in size to those observed by Choi et al. [20] (10–50 μm) in which iPSCs containing FAD mutations were embedded in Matrigel. Various conformation-specific antibodies that recognize different structural motifs on aggregated forms of Aβ have been widely used to characterise and classify oligomeric and fibrillar forms of Aβ [46,47]. The Aβ deposits formed on inhibition of IDE bound the conformation-dependent antibody A11, which recognizes prefibrillar oligomers, and antibody OC, which recognizes fibrillar oligomers as well as fibrils [32,33]. Aβ species recognized by the A11 and OC antibodies were present in the AD brain [48], although it was the fibrillar, OC-positive oligomers that correlated with the onset and severity of AD [49]. Thus, the Aβ deposits observed in the present study have immunological properties similar to those of Aβ aggregates present in the AD brain.

The inability of IDE inhibition to increase the number of Aβ deposits in the presence of the BACE1 inhibitor implies that the Aβ in the deposits is derived from ongoing proteolytic cleavage of APP. This is supported by the observation that IDE preferentially degrades monomeric rather than aggregated oligomeric forms of Aβ [37,50]. This attests to the dynamic nature of the ECM-encapsulated neuronal system in which Aβ production and degradation appear to be closely coupled. In the brain during AD, the build up of oligomeric forms of Aβ may diminish the role of IDE and increase the role of NEP, as NEP has been reported to be capable of cleaving oligomeric forms of Aβ [51].

In contrast with previous studies, where Aβ deposition in a 3D ECM was only observed when iPSC-derived neurons were expressing APP and/or presenilin 1 with familial AD associated mutations [20,52], the two iPSC lines used in the present study are derived from individuals without AD and were not genetically modified either to overexpress APP or with mutations in APP or the presenilins associated with familial AD. In contrast to previous studies [20,52] we did not see reproducible changes in the phosphorylation status of tau (data not shown). This may reflect the use in other studies of cells incorporating AD associated mutations that increased the Aβ42/40 ratio which tightly correlated with pathogenic tau accumulation and aggregation [52]. Although we also used Matrigel as the 3D ECM, it is possible that batch to batch variations in the Matrigel may also contribute to this difference. The use of alternative ECMs, such as porous silk protein sponges infused with collagen, may allow for more reproducible recapitulation of other pathological markers of AD including neuronal loss, reactive gliosis, neuroinflammation and diminished neural network functionality [53].

Overexpression of IDE in APP transgenic mice reduced brain Aβ levels and prevented Aβ plaque formation [54] and an IDE gene variant associated with elevated IDE expression was associated with reduced plasma Aβ levels and decreased risk of late-onset AD [55]. Such observations have led to activation of IDE being proposed as a therapeutic approach to AD (reviewed in [35]). The data presented here showing that IDE plays a major role in Aβ degradation in human iPSC-derived cortical neurons would support this approach. As IDE is implicated in the degradation of multiple β-forming peptides, including the peptide hormones amylin, atrial natriuretic hormone, glucagon and, of course, insulin, non-selective activation of IDE may have unwanted side-effects on normal physiological processes. However, allosteric modulation of the enzymatic activity of IDE towards pathological proteins, including Aβ, without affecting functional proteins like insulin has been proposed as a potential therapeutic strategy [56,57].

A limitation of our study is the use of neurons in isolation of other cell types present in the brain, particularly glia which are known to be involved in Aβ degradation and clearance [58]. Inhibition of IDE in co-cultures of neurons and glia within a 3D ECM would be required to address this point, although it should be noted that IDE is also the major Aβ-degrading enzyme in glia [36,59]. It would also be interesting to study the effect of IDE manipulation in co-culture models incorporating endothelial cells, pericytes and astrocytes alongside neurons [60] to determine the relative contribution of IDE and other Aβ-degrading proteases, together with transport across the blood–brain barrier, to Aβ degradation and clearance in a reverse-engineered 3D human neuronal model. As age is the largest risk factor for AD, a further limitation of our study is the use of iPSC-derived neurons which lack age-related changes. Although, as in the human brain neither IDE nor NEP activities change with age [61], the conclusions of our study are likely to hold. Our study used Aβ1-40 which is the major Aβ peptide present in the brain, is more soluble and less aggregation prone under the experimental conditions used here. Future studies should explore whether the longer, more aggregation prone Aβ1-42, which is also a substrate for both IDE and NEP [13,27], is also predominantly degraded by IDE in human neurons.

In conclusion, through the use of an Aβ degradation assay, selective protease inhibitors and iPSC-derived neurons, we have shown that IDE is the major contributor to Aβ degradation in iPSC-derived human neurons. By exploiting the observation that when cells are grown in a 3D ECM Aβ deposition can be visualised, we have shown that inhibition of IDE impacts Aβ metabolism in live neurons. This work supports strategies to enhance IDE activity to reduce Aβ accumulation as a potential therapeutic approach for AD.

## Data Availability

The data supporting the findings reported in the study are openly available from the University of Manchester FigShare repository doi: 10.48420/23998263.

## Open Access

Open access for this article was enabled by the participation of University of Manchester in an all-inclusive *Read & Publish* agreement with Portland Press and the Biochemical Society under a transformative agreement with JISC.

## Abbreviations

AD: Alzheimer’s disease
APP: amyloid precursor protein
Aβ: amyloid-β
BACE1: β-site APP cleaving enzyme-1
DAPI: 4′,6′-diamidino-2-phenylindole
ECE: endothelin-converting enzyme
ECM: extracellular matrix
IDE: insulin-degrading enzyme
iPSC: induced-pluripotent stem cell
NEP: neprilysin
NMM: neural maintenance medium
NPC: neural progenitor cell
PBS: phosphate-buffered saline
PFA: paraformaldehyde
